# Supplementary material for: Are carbon emissions trading and green financial instruments synergistic? -Comprehensive quantitative research based on content analysis
Source: PLoS One. 2024 Mar 7;19(3):e0298601. doi: 10.1371/journal.pone.0298601 (PMC10919601; doi:10.1371/journal.pone.0298601)
Supplement: S1 Data — (ZIP) [file pone.0298601.s001.zip › Data/地级市Z_F工作报告.zip!/╣π╢1⁄2/╘╞╕í╩╨/└┤╫╘╣1⁄2╓┌║┼í╛┬φ┐╦╩2╛▌═°í┐_2015.docx]

各位代表：

我代表市人民政府向大会作政府工作报告，请予审议，并请政协各位委员和其他列席人员提出意见。

2014年工作回顾

2014年，是我市面对复杂的经济形势，经济社会持续加快发展、重大项目建设成效显著、全面深化改革有序推进的一年。市政府在省委、省政府和市委的正确领导下，在市人大及其常委会和市政协的监督支持下，抓住省委省政府实施促进粤东西北地区振兴发展战略、佛山对口帮扶等重大发展机遇，迎难而上，积极作为，较好地完成了市五届人大四次会议确定的目标任务。

一、经济发展质量明显提高

预计全市实现地区生产总值674亿元，增长10.3%；人均生产总值增长9.6%；规模以上工业增加值260.28亿元，增长15.7%；地方公共财政预算收入52.85亿元，增长15.5%；固定资产投资总额742.43亿元，增长29%；社会消费品零售总额228.5亿元，增长12% ; 外贸进出口总额18.02亿美元，增长14%；城乡居民人均可支配收入14061元，增长10.4%；12月末，金融机构本外币存款余额826.3亿元、贷款余额533.29亿元，分别增长10.9%和13.1%。2014年，我市在省促进粤东西北地区振兴发展首次评估考核中,居粤北地区第一位。

（一）第一产业稳定增长。实现增加值148.1亿元，增长4.3%。粮食生产持续稳定，粮食播种面积达176万亩，总产量突破70万吨。农业产业化经营步伐加快，农业产业化组织达2425个，新增452个，其中县级以上农业龙头企业新增21家，农业产业化经营销售收入达562.8亿元，增长8.1%。温氏集团全年销售收入380亿元，在全国建成现代家庭农场2047个。新增广东省农业类名牌产品4个，“三品”认证16个，地理标志保护产品1个。罗定市苹塘镇稻田景观和泗纶镇分别被农业部认定为2014年中国美丽田园和第四批全国一村一品示范村镇。

（二）第二产业不断壮大。实现增加值299.7亿元，增长14.3%。全市规模以上企业达742家，增加123家，主营业务收入900亿元，增长27%，工业对经济增长贡献率达58%。“还珠工程”和企业成长工程深入实施，传统产业转型升级初见成效，产业结构进一步优化。石材、水泥、硫化工、不锈钢制品、电力五大产业产值分别实现318.3亿元、46.45亿元、40.85亿元、103.2亿元、72.9亿元；汽车零配件、生物制药、新型环保节能材料等新兴产业发展方兴未艾。中顺洁柔纸业、宏佳铝业、新合铝业、青洲水泥熟料生产线、发恩德矿业、贝融建材等一批重点工业企业项目建成投产。市石材机械产业标准联盟获省批准立项，填补全省在该领域标准的空白。

（三）第三产业快速发展。实现增加值226.2亿元，增长7.8%。房地产业和金融业、批发零售业、住宿餐饮业、信息传输业等服务业稳步提升。石材电子商务发展迅猛，中国石材第一网（云浮石材）年成交量达到2.8亿元，增长16%。以休闲度假和健康养生为主题的旅游业加快发展，接待游客1851万人次、增长37.31%，实现旅游总收入185亿元、增长23.39%。村镇银行、小额贷款公司等金融机构和组织有新突破。保险业加快发展，实现保费收入16.18亿元，增长23%。成功举办了第十一届中国（云浮）国际石材科技展览会暨第五届中国（云浮）石文化节。

二、经济发展后劲不断夯实

全年重点项目完成投资337.13亿元，完成年度投资计划89.55%。新引进合同项目162个，计划总投资246.2亿元。一批项目的建成和储备，极大地增强了经济发展后劲。

（一）交通基础设施不断完善。全市完成交通基础设施建设投入71.6亿元，增长9.82%。南广铁路正式开通运行。江罗、罗阳高速等重点交通设施建设进度加快，汕湛高速清远至云浮段和云浮至湛江段先行工程等项目动工建设，怀阳高速云浮段、云茂高速及沿江公路云浮都杨至牛远段、西江新城东部快线等工程的前期工作抓紧推进。积极与佛山等市联手推动共建机场工作，云浮罗定机场加紧盘活，西江航道云浮段和港口码头加快扩能升级改造。

（二）园区承载水平不断提高。全市重点工业园区累计入园项目达443个，投资总额703.81亿元，其中在建项目89个，计划投资226.63亿元，完成投资97.33亿元；建成投产项目278个，实际投资额282.48亿元。实现规模以上工业总产值350亿元，占全市比重35.5%；规模以上工业增加值100亿元，占全市比重36%。与佛山合作共建的佛山（云浮）产业转移工业园思劳片区启动建设，中意（云浮）产业园获中国中小企业国际合作协会授牌，规划和招商等工作抓紧进行。华为云计算数据中心等一批高端产业项目成功引进。

（三）城市扩容提质不断加快。部分行政区划调整顺利完成，云安撤县设区，云浮新区管委会获省政府批准成立。市中心城区公共服务设施进一步完善，蟠龙天湖改造、英东体育馆地下人防工程等加快建设，河滨西路、金丰路全线贯通，牧羊路主体工程改造顺利完成，金山路北段、永丰路南段等12条市政道路建设抓紧推进。全市完成“三旧”改造项目62个，改造面积1415.35亩。创建国家卫生城市通过检查验收，成功创建省文明城市。顺利发行第二期“粤云浮债”，募集债券资金10亿元。云浮新区核心区—西江新城建设有序推进，建成市政道路14条21.1公里，同步配套完善供水、排水、供电等市政设施，碧桂园城市花园、远大美域、宝能特色、云浮印象中式大院等项目建设加快，碧桂园国际学校、远大酒店、领美酒店等配套项目抓紧推进。广东华立城市学院扩大招生，华南师范大学附属云浮学校已签约。云安、罗定、新兴、郁南的城市功能不断完善，市容市貌焕然一新。

三、加快发展活力有效激发

（一）行政体制和经济领域改革不断深化。全市共调整压减行政审批事项1569项，调整压减率达45.02%，提前实现省提出的目标。在去年全省促进粤东西北地区振兴发展行政审批制度改革评估考核中，我市得分列全省第一名。网上办事大厅开通至镇，比省要求提前3年，目前已进驻事项1195项，审批事项进驻率100%。建设公共资源交易中心，政府采购节约率9.05%，建设工程交易项目中标价平均下浮率11%。商事制度改革全面推进，市场主体增量提质，全市874户个体工商户升级登记为企业；新登记市场主体1.29万户，实有市场主体7.29万户、增长9.74%。“品牌云浮”建设积极推进，新获认定广东省著名商标9件，9家企业的9个产品获得广东省名牌产品称号。大力推进市县政府机构改革，发改、商务、卫生计生、食品药品监管等部门的管理体制调整工作顺利完成。严厉打击走私、制假售假违法活动，维护了良好的社会经济秩序。

（二）农村综合改革稳步推进。在全省率先建立市县两级征信中心，修订了全省首个农户信用标准，农户信用信息系统为全市57.62万户家庭全部建立信用档案，19.59万农户被评定为信用户，建成信用村841条。农村普惠型金融体系建设加快推进，粤东西北地区农村金融工作现场会在云浮召开，成为广东省社会创新实践基地、社会信用体系建设试点市、农村金融改革创新综合试验区。在去年底的全国农村改革试验区工作交流会上，汪洋副总理充分肯定了我市现代农业经营体制机制创新的经验和做法。

（三）社会创新治理初见成效。全市登记的社会组织697个，其中新登记社会组织99个；备案的社会组织8203个，备案登记率100%。我市培育发展自然村乡贤理事会经验在全国“探索村民自治的有效实现形式高端研讨会”上推广。取消对全市性社会团体分支（代表）机构设立、变更、注销的登记和备案，简化群众办事程序。城乡社区自治和服务功能持续增强，全市847个村民委员会、118个社区居委会圆满完成“两委”换届选举工作。

四、社会民生福祉持续提升

全市民生类支出完成86.11亿元，增长13.07%，占公共财政预算支出的69.47%。

（一）生态文明工程扎实推进。新一轮绿化广东大行动深入开展，完成造林面积14.74万亩，乡村绿化美化建设村庄136条，全市森林覆盖率68.9%。累计投入建设资金7.8亿元，创建生态文明村2745条。21个示范镇的总体规划和控制性规划基本完成，5个宜居城镇、77条宜居村庄、5个宜居社区建设有序推进。建成广东名镇5个、广东名村13条，省立绿道90公里。完成省下达的农村生活垃圾治理任务，全市8788条自然村均建成符合省要求的垃圾收集点，农村生活垃圾无害化处理率70.93%。切实抓好环境保护，全市356个减排项目全部完成，四项主要污染物总量减排完成省下达任务。城乡水环境和空气质量监管进一步强化，饮用水源水质100%达标。城镇生活污水集中处理率74.5%（其中市区82.5%）。耕地保护工作成效显著，通过省政府2013年度耕地保护目标责任制履行情况考核，连续14年实现耕地占补平衡。

（二）社会保障和就业工作稳步推进。全市常住人口社会保障卡持卡率98.21%，基本实现全覆盖。城乡居保基础养老金标准提高到每人每月80元，城乡居民医保住院医疗费政策报销比例达到70.5%。底线民生保障水平稳步提高，城乡低保分别比省提出的第四类地区最低标准高20元，达到338元和156元。孤儿基本生活最低养育标准集中和散居分别比上年提高150元和100元。城乡低保对象基本医疗救助比例达到70%，实现“一站式”结算服务。农村五保供养水平达到上年人均纯收入的60%。对全市所有低保、五保、“三无人员”等困难群体参加城乡居民基本医疗保险进行全额补助，并为全市低保对象和70岁以上老人购买了人身意外伤害保险。全面落实各项就业扶持政策，新增城镇就业35582人，失业人员再就业9327人，新增农村劳动力转移就业36325人，组织劳动力技能培训30626人，全市城镇登记失业率2.46%。创建和谐劳动关系示范区工程示范点48个，涉及企业总数662户、职工94464人。

（三）民生各项事业统筹推进。市政府承诺办好的“十件民生实事”全面完成，解决了教育、医疗、住房等领域的一批热点难点问题。教育创强顺利推进，规划创建的16个镇（街）通过省督前检查，云城、新兴创建教育强县（区）及义务教育均衡县（区）进展顺利。全市学前教育毛入园率90.68%。云浮终身学习网通过省教师远程培训平台评估。市技工学校通过高级技工学校评审，云浮恒大高级中学、臻汇园小学动工建设。科技创新能力增强，全市专利申请610件，增长16.2%；专利授权437件，增长3.6%；新建 2 个省级创新平台。医疗卫生服务水平显著提升，城乡居民规范化电子健康档案建档率96.2%。县级公立医院综合改革全面推开，试点医院实现药品“零差率”销售。市人民医院、市中医院、罗定中医院成功创建“三甲”医院。有效防控登革热和人感染H7N9禽流感等传染病，全市无重大传染病疫情暴发流行和突发公共卫生事件发生。人口与计划生育工作完成年度目标管理任务，低生育水平持续稳定。体育文化实事件件落实，34个镇（街）成立了全民健身服务站，建成13个镇（街）农民体育健身工程、9个社区体育公园，成功举办了首届“市长杯”足球联赛、2014年国际女子沙滩排球精英赛；建成镇（街）综合文化站和行政村（社区）文化公共电子阅览室110间、村落历史文化展示馆10个；第一次全国可移动文物普查和海上丝绸之路南江古道调查工作顺利开展，对磨刀山遗址进行了考古发掘。扶贫开发“双到”工作取得新成效，累计投入帮扶资金4.28亿元，其中佛山对口帮扶累计投入2.2亿元，99个重点帮扶村实施帮扶项目3019个。完成3365户农村低收入住房困难户住房改造建设任务，动工建设保障房项目7个，基本建成775套。超额完成省推广应用LED照明产品工作（粤东西北地区）任务，改造公共照明（路灯、景观灯）4.28万盏。中小河流治理、病险水库除险加固、村村通自来水工程、省级小型农田水利重点县、中小型灌区续建配套与节水改造等民生水利工程建设加快，累计完成投资4.66亿元。平安云浮建设深入推进，“六大专项”打击整治行动成效明显，视频监控系统新增3199个高清一类治安监控点、79个高清治安卡口及37套高清电子警察，各类违法犯罪警情下降3.9%。认真做好矛盾化解工作，市、县两级受理群众来信来访件（人次）下降20.55%，没有发生影响恶劣的重大刑事案件和重大群体性事件。组织打击“三违三抢”行动103次，依法强制拆除违法建筑30万平方米，清理抢种青苗31万株。深入落实安全生产“一岗双责”，安全生产和食品药品安全形势持续稳定，没有发生较大以上生产安全事故和重大食品药品安全事故。

五、政府自身建设全面增强

（一）民主法治进一步推进。认真执行市人大及其常委会的决议、决定，主动接受市人大的法律监督、工作监督和市政协的民主监督，坚持重大事项向人大报告、向政协通报，人大代表建议和政协委员提案办理答复率和满意率均为100%。认真听取各民主党派、工商联、无党派人士、人民团体和社会各界人士的意见。大力推进依法行政，制定政府规范性文件14件。严格执行市政府常务会议工作规程和重大决策专家咨询评估制度。

（二）工作作风进一步转变。扎实开展党的群众路线教育实践活动，严格落实中央“八项规定”和“约法三章”，认真整治“四风”存在问题。建立健全市政府工作部署、监督落实、督查反馈等工作制度，坚持重大项目、重点工作责任制，在有效化解立项报批、项目用地、企业用工、征地拆迁等难题上进行了大胆的探索。强化绩效管理，完善考核指标体系，提高政府工作效能。重视基层和群众意见，认真整改，转变作风，努力解决服务群众“最后一公里”问题。

（三）廉政建设进一步加强。认真落实政府部门党风廉政建设责任制，严格履行“一岗双责”。进一步建立健全惩治和预防腐败体系，加强了对权力运行特别是行政审批、政府采购、项目建设、公共资源交易等重要领域的行政监察和审计监督。坚持厉行节约、勤俭办事，严格控制财政供养人员，压减了一批节庆、论坛活动，会议费和“三公”经费同比下降21.83%。

此外，积极贯彻依法治军、从严治军方针，军分区部队和民兵预备役建设稳步发展。统计、物价、档案、外事侨务、港澳事务、台务、人防、防震减灾、地方志、民族宗教、消防、保密、气象、工会、青年、妇儿、侨联、文联、社科联、工商联、贸促会、代建服务、新闻出版、红十字会以及驻市外机构等工作也取得了新成绩。

各位代表！过去一年，我们面对繁重的改革发展任务，取得的成绩来之不易，这是全市广大干部群众共同拼搏的结果。在此，我代表市人民政府，向全市各条战线上的干部群众，向人大代表、政协委员，向各民主党派、工商联、无党派人士、人民团体，向离退休老领导、老同志，向驻云浮部队、武警官兵、公安干警，向所有关心支持云浮发展的社会各界人士，致以崇高的敬意和衷心的感谢！

在肯定工作成绩的同时，我们也清醒地认识到，工作和发展中还存在一些深层次的问题，主要是：土地、资金、人才等要素制约日显突出，加快发展难度进一步增大；“量小底薄”，经济基础还不牢固，发展质量有待进一步提升；财政和城乡居民持续增收还不够快，民生问题有待进一步解决；节能减排与经济快速发展的矛盾依然突出，环境保护压力进一步加大；有些单位和公职人员执行力不够强，一些工作落实还不到位，部分重点项目建设推进较慢；政府职能有待进一步转变，廉政、效能建设有待进一步加强；等等。对这些问题，我们必须高度重视，采取有力的措施认真加以解决，决不辜负全市人民的期望！

2015年工作安排

各位代表，2015年是全面深化改革的关键之年，是全面落实依法治国方略的开局之年，也是全面完成“十二五”规划的收官之年，做好今年的工作意义重大。习近平总书记关于我国经济发展新常态的一系列重要论述，为我们指明了前进的方向。当前，中央坚持稳增长、促改革、调结构、惠民生、防风险的一系列重大举措进一步落实，国家实施“珠江—西江经济带”规划，省大力促进粤东西北地区振兴发展，构建“广佛肇+清远、云浮” 新型大都市圈，佛山市对口帮扶工作深入推进，我市迎来的机遇前所未有。我们必须乘大势，抓机遇，奋发有为，全力开创改革发展新局面。

今年工作的总体要求是：全面落实党的十八大、十八届三中、四中全会、中央经济工作会议、省委十一届四次全会和市委五届六次全会精神，主动适应经济发展新常态，牢牢把握“跨越赶超、科学崛起”主题，坚持生态立市、产业兴市、特色美市、改革活市，狠抓招商、建城、改革，加快产业转型升级，推进城市扩容提质，不断优化发展环境，实现经济快速健康发展和社会和谐稳定，全面建设现代生态城市。

今年的主要预期目标是：地区生产总值增长12%左右；人均地区生产总值增长11.3 %左右；地方公共财政预算收入增长16%；规模以上工业增加值增长20%；固定资产投资总额增长25%；社会消费品零售总额增长12.5%；居民消费价格涨幅在1.8%左右；外贸进出口总额增长7%；城乡居民人均可支配收入增长8%；完成省下达的节能减排任务。同时安排79项重点工程（项目），计划总投资1072.56亿元，年度计划投资188.39亿元。

为实现上述目标,必须做到方向不偏、科学谋划、突出重点、持续用力。着重抓好以下五个方面工作：

一、坚定不移建设现代产业体系

围绕“四新一特”产业，努力“招大、引强、选优”招商，提升传统产业，做大做强实体经济，实现产业兴市。

（一）加快发展现代工业。

突出发展战略性新兴产业。大力发展云计算及信息服务产业。加快与华为公司合作建设云计算数据中心，以龙头项目带动云计算产业发展取得突破。规划建设“云谷”，把其建成电商区域性总部基地、大数据处理产业基地、信息服务及外包产业基地、数字媒资产业基地，打造华南领先乃至全国有名的云计算产业集聚地。加快智慧城市建设，稳步推进三网融合，促进物联网研发和示范应用，提升软件服务、网络增值服务等信息服务能力。鼓励支持电子商务新业态发展，深入拓展网上商城、网上政务、电信增值等行业服务。大力发展生物制药产业，依托大华农、远大药业，吸引生物医药产业集聚发展。积极培育生物育种产业，推广绿色农用生物产品，促进生物农业加快发展。坚持发展节能环保产业和新型环保建筑材料，提高资源综合利用水平和再制造产业化水平。大力发展新能源，加快建设适应新形势发展的智能电网及运行体系。加快建设中小企业孵化器和创新产业化示范基地，营造扶持高新技术企业发展的良好氛围。

优先培育壮大现代制造业。抓住省打造珠江西岸先进装备制造产业带的重大机遇，制订先进装备制造业中长期规划，推动我市融入珠江西岸先进装备制造产业带。大力鼓励工业自动化、智能化技术创新，打造一批集研发、设计、制造、营销、安装和售后服务等为一体的装备制造服务企业，推动我市装备制造业由粗放加工向精密制造和工艺创新转变。重点发展汽车零配件、环保设备、石材装备产业，加快形成一批数字控制技术、计算机技术、电机技术等高新技术产业。加快整合石材机械装备制造产业资源，深入推进石材机械产业标准化工作。推进科技创新成果转化应用，为提高生产效率和产品质量提供支撑。

持续做大做强传统产业。实施工业转型升级攻坚战3年行动计划，重点培育一批龙头企业和更多规模以上工业企业。大力推动新一轮企业技术改造，突出石材、水泥、不锈钢制品、硫化工等传统产业的补链强链，加快产业转型升级。石材产业方面，推动产业链向文化创意、工程设计、精细化等方向发展，擦亮云浮石材世界级品牌。水泥产业方面，推动产业链从水泥熟料向水泥成品与水泥深加工延伸。不锈钢产业方面，引导不锈钢制品企业抱团发展，加强品牌培育，促进不锈钢制品向高端产品转型。硫化工产业方面，加快引进和研发先进生产技术，深度开发硫化工产品，不断提高附加值。继续加快华润西江发电厂、粤泷电厂扩能改造等电力项目建设，争取云浮电厂D厂获批上马，促进电力产业化发展。加大对重点大型骨干企业的培育和帮扶力度，继续推进“还珠工程”、企业成长工程，促进中小微企业和个体工商户转型升级，年内重点帮扶一批高成长性中小企业（民营企业）发展，着力培育100家规上工业企业、100家限上商贸企业。

（二）加快发展现代农业。

积极实施粮食稳定增产行动，大力推进农业综合开发，不断完善农田水利设施，建设12.48万亩高标准基本农田。全面启动农村土地承包经营权确权登记颁证工作，规范引导农村土地经营权有序流转。加快转变农业发展方式，培育农业产业化龙头企业、现代家庭农场、现代农民合作社等新型农业经营主体，发展多种形式的农业适度规模经营，重点建设5大现代农业产业园区。力争年内新发展省级农业龙头企业2家，市级农业龙头企业8家,新增“三品”认证20个。提升农产品质量安全水平，深度开发健康食品生产加工业，把我市建设成全国生态农业发展的标杆城市和示范基地。深入推进全国农村改革试验区建设，不断激发农村经济发展活力。加快云浮市省级新农村示范片项目建设。加快推进省农村普惠金融“八项行动”，积极推进涉农保险，探索建立农村产权交易平台，全面增强金融服务“三农”发展能力。推动南粤银行云浮市分行筹建和云城、云安、罗定、郁南农信社改制农商行工作，实现村镇银行和农业特色小额贷款公司在县域全覆盖。深入推进信用村、信用户建设，扎实开展信用示范村创建活动。

（三）加快发展现代服务业。

增强会展经济意识，充分发挥好中国·云浮国际石材产业城、云浮国际石材博览中心和石材长廊的效应。继续推进流通现代化，加快云浮新港物流园、珠钢星晖码头等物流园区建设。依托云浮新港优势，在云安区试点探索金融物流新模式。积极培育新兴消费热点，推进云浮产品广货网上行活动。支持规划建设罗定汽车贸易园区项目。大力发展资本市场，鼓励各类企业通过多层次资本市场开展直接融资，确定和培育20家上市后备企业，推进温氏集团整体上市。鼓励发展审计评估、金融中介等中介服务业、工业设计和服务外包、文化创意等产业。推进“万村千乡”市场体系建设，促进服务业与制造业、现代农业融合互动发展。积极建设“全国智慧旅游试点市”，推动广东禅文化创意产业园、石艺文化旅游产业园和南江文化旅游产业园加快发展，开发富有云浮特色的健康服务产业和休闲度假产品，打造全国知名的健康产业高地、休闲养生目的地。

二、坚定不移构筑加快发展平台

加快推进战略性发展平台建设，明确主攻方向，打造新的发展引擎，对增强发展后劲、加快云浮发展促转型具有重要的战略意义。

（一）加快西江新城建设。

西江新城是云浮新区的核心区，是我市未来的政治、经济、教育、文化中心。我们要积极担当，不辱使命，坚定不移把新城谋划好、建设好、发展好。

加快完善城市基础设施和提升环境质量。重点抓好南广铁路交通枢纽、中央商务区、新城快线、东部快线、北湖启动区、特色小镇等项目建设。加快推进广东华立城市学院综合项目、华南师范大学附属云浮学校、碧桂园国际学校、污水处理厂、11万伏和22万伏变电站，以及公办学校、医院、保障房和商业街、美食街等配套设施建设。加快推进东山、凤凰山森林公园以及水景中轴线的规划建设，提升新城的城市品位。

坚持产城融合提升集聚能力。推动云安的商贸金融、文化教育、医疗卫生、居住、行政服务等功能加快向西江新城集中。充分发挥新城的生态和资源优势，引进一批科技含量高、带动能力强、税收效益好、无污染的高新技术项目，以及商贸物流、教育学校、休闲旅游、健康养生、体育运动等城市产业项目。不断优化融资结构，创新投融资方式，为新城加快发展提供良好的资金保障。大力推进新城公共服务体系建设，加强社会管理综合治理，增强人民群众对云浮新城的认同感、支持度。

（二）加快工业园区建设。明确各重点园区和集聚区的发展定位和产业重点，加快形成主导产业。佛山（云浮）产业转移工业园都杨片区及思劳片区要在产业质量和发展效益上有新突破；六都片区、双东片区、都城片区要争取尽快纳入佛山（云浮）产业转移工业园产业集聚地；佛山顺德（云浮新兴新成）产业转移工业园要在扩能增效上有所突破。中意（云浮）产业园要进一步完善发展规划和产业规划，制定投资优惠办法，落实项目建设用地，努力吸引意大利和其它地区的优势产业项目转移至园区发展。力争今年全市重点产业园区实现工业总产值600亿元、工业增加值150亿元。其中，实现规模以上工业总产值500亿元，占全市比重40%。

（三）加快合作平台建设。充分利用佛山对口帮扶的有利契机，加强产业合作，有针对性地引入一批大项目、好项目。深化两地在农业、金融、科技、人才、文化、旅游、体制改革等方面的合作，启动和实施两市教育、卫生千人互派和名校、名医院交流合作计划，实现优质资源共建共享。在《珠江—西江经济带发展规划》实施中寻求对外交流合作新突破，大力推动新兴、罗定、郁南与周边地区的经贸合作与交流。完善网络招商信息平台建设，升级“云浮招商网”，推进招商引资深度开展。积极采取灵活务实的招商方式，举办行业展会和有针对性的小型对接会，积极开展小分队招商，提高招商引资实效。健全大项目跟踪台账制度，提高项目履约率和落地率。

（四）加快创新服务平台建设。加快社会信用体系和市场监管体系建设，积极推进投融资机制创新，激发发展潜力。建设科技金融服务中心，搭建科技金融服务平台。支持和引导科技产业基金、科技风险投资和信贷融资发展。充分发挥金融互联网、物联网的平台作用，拓展石材网上推广和交易力度。发挥国家石材产品质量监督检测中心的公共技术服务平台作用，不断提升石材产品质量。加快中小企业服务体系建设，完善一批功能齐备的中小企业服务平台。加快企业技术研究开发中心和工程中心等创新服务平台建设，推进产学研深度合作。培育和壮大一批高新技术企业，争取年内3至5家获高新技术企业认定。加大云浮高新区孵化器建设力度，引导科技型中小微企业开展自主创新。设立政府引导资金，推动政银企合作，试点开展“助保贷”业务，破解中小企业融资难问题。

三、坚定不移推进城乡统筹发展

以现代生态城市为引领，突出抓好城乡建设，大力构筑便捷高效的综合交通运输体系，强化环境保护和科学利用资源，推动城乡区域互促共进。

（一）着力提升新型城镇化体系。

加快拓展现代生态城市发展空间。抓住省构建“广佛肇+清远、云浮”新型大都市圈机遇，加快建设现代生态城市。深度融合云浮建设“全国农村改革试验区、全省循环经济和人居环境建设示范市、生态文明建设示范区”等的功能定位，重点规划构建“一核三副六廊”的市域空间格局，与中心镇以及一般建制镇共同组成多层次的城镇服务中心体系。努力完善与新型大都市圈相匹配的交通网络、产业体系、城市配套等基础设施。研究和制定佛山云浮两市一体化发展规划，打造两地在交通、产业、城市、国资企业、政务营商环境及人才流动等全方位、宽领域、多层次区域一体化发展新格局，实现优势互补、发展共赢。

加快提升“一核”城市承载力。充分利用行政区划调整的契机，做大做强以云城城区、西江新城、六都、思劳“四组团”构成的中心城区，着力解决新老城互动和产城融合“两大问题”，使其成为加快发展的主引擎和增长极。不断深化城市规划和设计，夯实中心城区的地理信息和规划信息基础，推动精细化城市建设管理，构筑现代化功能型的城市基础设施和服务设施体系，全面提升城市功能和品位。以建设精品生活服务轴和高端产业联系轴为纽带，加快推进新城和老城的交通、供电、供水、供气等市政设施建设，完善教育、医疗、文化、体育等公共服务设施。以打造核心圈层、中圈层、外圈层为联结，促进产业与城市发展的深度融合。继续抓好城区道路贯通工程，完善城市路网结构。不断增加城区停车场和公共广场、绿化等开敞空间，改善交通出行环境和城市环境。逐步把云城城区石材企业搬迁到石材园区。

加快建设“三副”城市步伐。支持罗定、新兴、郁南三个城市副中心的扩容提质，推动罗定泷洲新城、新兴特色园区和郁南九星新城建设，不断扩大城区面积和完善城市功能，增强城市吸引力。利用云浮山水特色和人文传统，深入开展小城市和特色小城镇的培育工作，依托小城镇和中心村打造功能完善的农民生活服务圈，全面增强现有中心镇、专业镇的衔接城乡功能，促进人口、产业集聚发展。同时，稳步开展户籍制度改革，推动农村富余劳动力向城镇转移落户，有序推进转移人口市民化。加快城乡统筹协调发展的综合配套改革，建立城乡优势互补、良性互动的发展格局，促进城乡基本公共服务均等化，实现城乡融合、协调发展。

（二）着力构建全方位的大交通体系。

全面推进水陆铁空交通建设，加快构筑“六廊”通道。全年交通计划投资73亿元。公路方面，重点加快江罗高速、罗阳高速、汕湛高速云浮段等在建项目建设，完成江罗高速通新兴县、新城快线一期项目，完成全省“十二五”县县通高速的目标任务；动工建设鹤开高速新兴段；积极做好怀阳高速、云茂高速、广云高速扩建的前期工作；推动云浮新区互通立交、思劳立交东移、高村立交连接线建设。完成综合运输体系发展“十三五”规划、公共交通规划及公路网修编工作；抓好省道368线彩营至大河路段路面改造等公路迎“国检”项目改造工作，确保完成目标任务；争取将一批省道、县道和地方公路纳入升级改造计划，提升公路等级；建设农村公路420公里；建立“以西江新城、云城城区为中心，云安、郁南、罗定和新兴为依托，辐射周边镇街”的城乡一体化大公交网络，逐步实现公交线路与新规划的公路、铁路站场无缝接驳。铁路方面，加快罗岑铁路股权回收并争取纳入国家铁路网，争取推进广茂铁路升级改造的前期工作。水路方面，加快西江3000吨级航道扩能升级整治及南江口码头扩能等项目建设。航空方面，利用云浮罗定机场，推动建设通用航空产业经济园；推动与佛山等市共建机场的规划建设。

（三）着力构建良好的生态环境体系。

坚持绿色生态发展。深入实施生态景观林、森林碳汇、森林进城围城和乡村绿化美化四大林业重点生态工程。加强森林资源保护，扎实推进“三江五道”第一重山的生态保护及林地的生态功能保护。尽快消灭全市现有的1.5万亩宜林荒山，改造2.2万亩疏残林、纯松林和布局不合理的速生林，推进4.2万亩新老城区林相改造。全面实施加强环境保护、推进生态文明建设“三年行动”计划，加快罗定第三污水处理厂和腰古、南江口、富林等镇以及郁南大湾园区等地的污水处理厂建设运行，加快生态文明示范镇建设、幸福村居、“三个宜居”、农村生活垃圾处理及农村泥砖房改造等工作。全面划定生态控制线，坚守生态环保红线，强化环境分区控制，实施差别化环境保护准入和最严格的源头保护制度。严格环评审批监管，确保所有新建项目落实环评和“三同时”制度。纵深推进农村饮水安全、污水垃圾处理等环境综合整治工作，加快迳尾水库备用水源引水工程建设，保障市城区应急供水安全；规范禽畜牧业养殖，严厉打击偷排放、乱排放污水污物行为，切实保护地下水资源安全。

坚持推进节能减排。继续强化节能目标责任管理，加大对列入市节能预警地区的监控和督导，坚决拒绝高耗能重污染项目。全力推进粉尘综合防治、电厂陶瓷厂脱硫脱硝、锅炉污染治理、油气回收改造等大气污染治理工程，以及采矿、硫化工等重点行业的重金属污染治理。积极推行石材、建材、纺织印染、化工等行业的清洁化生产和建筑、交通、公共机构等非工领域的节能，坚决淘汰现有企业的落后产能，严控企业的能耗增长和超能耗限额运行。坚持标本兼治的原则，从快从严查处企业超标排污和偷排放行为。全面完成2005年底前注册营运的“黄标车”淘汰工作。全力整治城区及周边粉尘污染源，力争颗粒物年均浓度比上年下降。加强PM2.5 、PM10的监测，力争全年空气优良率90%以上。

坚持节约集约利用资源。加强耕地保护，严格实施年度土地利用计划。加强闲置土地清查处置，盘活低效用地，做大土地储备总量。修编完善“三旧”改造规划，不断健全“三旧”改造的相关配套政策，借鉴和探索多模式、多类型的“三旧”改造措施。充分用活用好城乡建设用地增减挂钩政策，加快原云浮钢铁厂、粤云新型石材厂等一批中心城区的“三旧”改造项目建设。坚持节约集约利用土地，提高项目用地准入门槛，坚决打击以企业项目名义圈地、屯地、占地行为。大力推进水土流失综合治理，抓好水资源的节约保护。进一步规范河砂开采，合理开发矿产资源，坚决打击偷采滥采河砂和矿产资源行为。继续保持打击“三违三抢”行为的高压态势。

四、坚定不移推进社会事业建设

今年继续重点办好“十件民生实事”，统筹推进社会各项事业发展，让发展的成果惠及全市广大人民。

（一）积极实施就业创业工程。落实更加积极的就业政策，健全促进就业创业体制机制，促进高校毕业生、农村劳动力、就业困难人员等重点群体就业。探索建立经济发展和扩大就业更加紧密的联动机制，进一步创造就业机会，新增城镇就业3万人，转移农村劳动力2.9万人以上。落实激励创业的各项政策，加强基层公共就业服务平台建设，加快在线人力资源市场和人事考试基地建设。大力开展职业技能培训，实施农村劳动力、被征地农民、新一轮扶贫开发等专项培训工程。

（二）加快实施社会保障全覆盖工程。扩大社会保险覆盖面，引导城乡居民参保续保。做好被征地农民的社会保障工作。深入开展社会保险法执法检查，促进企业全员足额参保。扩大政策性农业保险范围，探索提供农民因灾损毁房屋保险服务。改造农村低收入住房困难户住房3420 户，建设城镇保障性住房2105套，基本建成1000套。推进公租房和廉租房并轨，探索把农业转移人口和外来务工人员纳入住房保障范围，解决新就业群体阶段性居住问题。加强城乡低保、特困人员供养、社会福利、养老等工作。每个县（市、区）建设1个城市社区居家养老示范点。加快建立各种社会保障衔接和统一的社会救助体系，健全托底性的社会保障制度。创新扶贫开发方式，实行精准扶贫，优化整合扶贫资源，确保完成第二轮三年扶贫攻坚任务。

（三）全面完成教育强市工程。年内全市新增教育强镇（街）7个，云安、罗定、郁南全面创建成为广东省教育强县（市、区），公办义务教育标准化学校100%，省教育强镇覆盖率100%，把云浮创建成教育强市。持续推进教育信息化均衡发展，基本实现“三通”，初步建成市级教育管理、教育资源公共服务平台。加强教师队伍建设，深入实施强师工程。进一步加大教育投入，完善家庭经济困难学生资助体系，促进教育公平。加快推进开放大学建设，推动社区大学深入开展。继续提升罗定职业技术学院、新兴中药学校、邓发纪念中学、云浮中专和市技工学校的办学质量。大力发展民办教育，加快推进华南师范大学附属云浮学校、云浮恒大高级中学建设，推动民办教育品牌化发展。

（四）大力实施公共文化培育工程。深化文化体制改革，增强文化创造活力，推动文化产业快速发展。构建公共文化服务体系，促进城乡基本公共文化服务均等化。加强城乡文化体育基础设施建设，推动公共文化设施向社会公众开放。推进省、市级公共文化服务示范区（项目）建设。加大文物保护力度，坚持扫黄打非，强化文化市场监管。加快发展创意、动漫、数字等新型文化业态。挖掘云浮本土文化遗产，打造本土文化精品，继续提升禅宗文化、石艺文化、南江文化等文化品牌影响力。深入开展全民健身活动，促进群众体育和竞技体育同步、全面发展。

（五）推进实施卫生惠民工程。加快推进以县级公立医院为重点的公立医院综合改革。巩固扩大基本医疗保障覆盖面，确保全市各项基本医疗保险参保率98%以上。建立健全重大疾病医疗保险和医疗应急救助制度，扩大城乡居民大病保险范围。完善卫生计生服务体系，加快建设市人民医院二期工程、市妇幼保健院改扩建项目和中央投资卫生项目。加强镇（街）卫生院建设，实施医疗设备“五个一工程”。继续推进中医药强市建设，做好全国基层中医药工作先进单位的复审。继续巩固创国卫和省文明城市成果。加强人口与计划生育工作，落实层级动态管理责任制和“一票否决”制度，确保人口出生率和自然增长率控制在省下达的指标内。落实“单独两孩”等国家调整完善的有关计划生育政策，做好计划生育家庭意外伤害保险和失独、独生子女伤残家庭帮扶工作。

（六）加快实施“平安云浮”工程。进一步加强“平安细胞”工程建设，构建社会治安立体化防控体系。巩固深化“六大专项”行动成果及行之有效的机制，持续开展打击涉枪、涉毒、“两抢一盗”等整治行动。健全重大决策社会稳定风险评估机制、突发事件应急处置机制。整合资源，打造全方位、多层次的反走私综合治理平台。加强互联网监管，强化网络舆情引导和管理。加强安全生产和食品药品监督管理，防范重特大安全事故和食品安全事故发生。加强学校、医院安全管理，创建平安校园、平安医院。全面推进以城市住宅小区和农村为重点的协同共治体系建设。

五、坚定不移加快建设法治政府

深入推进依法行政，加快建设职能科学、权责法定、执法严明、公开公正、廉洁高效、守法诚信的法治政府。

（一）深入推进民主法治领域改革。继续抓好法治文化建设，积极开展“六五”普法。坚持用法治方式推进社会综合治理和基层民主法治建设。加强农村集体“三资”监管，构筑农村“三资”管理服务平台，建立健全村务监督委员会，建设廉洁诚信镇村。建立健全社会矛盾预警机制、群众利益表达机制、协商沟通机制，畅通群众利益协调、权益保障法律渠道，引导群众依法表达利益诉求，切实解决“信访不信法”问题，依法保障群众合法权益，做到法治为民。加强法律援助，全面开展一村（社区）一法律顾问工作，进一步提高公共法律服务水平。

（二）深入推进服务型政府建设。进一步转变政府职能，加快行政审批制度改革步伐，提高行政审批效率。全面推进商事制度改革和行政审批流程优化再造及标准化建设。深入推进清理行政职权、编制权责清单工作，公布市直部门和县（市、区）部门权责清单。完善政务服务体系，继续推动网上办事大厅和基层便民服务平台建设，让更多的行政审批事项和公共服务事项在网上办理，逐步实现网上办事全流程、全天候、全地域覆盖和“零障碍”。改善窗口服务环境，为群众提供更加集中、便捷、高效的政府服务。

（三）深入推进依法行政。强化法治理念、法治思维和规则意识，坚决杜绝“以言代法、以权压法、徇私枉法”。推进行政执法信息化建设和信息共享，加大行政审批、行政处罚以及政府预算决算、部门预算决算、财政审计结果等的信息公开力度，让权力在阳光下运行。自觉接受人大法律监督和政协民主监督，认真执行人大及其常委会的决议决定，及时办理人大代表议案、建议和政协委员提案。认真听取各民主党派、工商联、无党派人士和人民团体的意见建议。进一步巩固党的群众路线教育实践活动成果，坚持以问题为导向，加大专项整治力度。继续落实中央“八项规定”和《党政机关厉行节约反对浪费条例》，完成公务用车制度改革，严控“三公”经费支出。完善惩治和预防腐败体系，规范征地拆迁和强农惠农资金的监管，加强行政监察和审计监督，坚决惩治腐败，建设廉洁政府。

同时,我们还要继续加强国防动员、国防教育和国防后备力量建设；充分发挥工会、共青团、妇联等群团组织的作用，依法保障公民特别是妇女、未成年人和残疾人的合法权益；继续做好外事侨务、港澳、对台、民族、宗教、贸促会、工商联、社科联、侨联、文联、人防、气象、防震减灾、水文、档案、地方志、代建服务、红十字会以及驻市外机构等工作。

各位代表！面对新形势、新任务，我们深感使命光荣和责任重大，加快发展、造福人民是政府施政所向！让我们在中共云浮市委的坚强领导下，齐心协力，开拓进取，扎实工作，为建设现代生态城市，让全市人民过上更加美好幸福的生活而努力奋斗！
